# Utilizing Population Distribution Patterns for Disaster Vulnerability Assessment: Case of Foreign Residents in the Tokyo Metropolitan Area of Japan

**DOI:** 10.3390/ijerph18084061

**Published:** 2021-04-12

**Authors:** Bismark Adu-Gyamfi, Rajib Shaw

**Affiliations:** Graduate School of Media and Governance, Keio University (SFC), 5322 Endo, Fujisawa-shi, Kanagawa 252-0882, Japan

**Keywords:** migrants, disaster vulnerability, population dynamics, Tokyo metropolitan area

## Abstract

Foreign residents in Japan are amongst the vulnerable groups at risk to disasters in the country. Improvement is crucial in meeting Japan’s vison of zero casualties in major disaster events. If the case of the 2005 Hurricane Katrina is to offer an insight into migrants’ characteristics in mega-disaster situations, then a broader analysis of vulnerabilities is needed to avoid mass casualties should the anticipated megathrust earthquake occur. Hence, this study analyzes the vulnerabilities of foreign residents by utilizing their spatial distribution attributes in the Tokyo metropolitan area. This study uses multiple approaches that combine geographic information systems to analyze secondary and primary datasets. The results reveal that foreign nationals are spatially clustered in some parts of the metropolis, especially within a 7 km radius of Minato city. The densities in these areas alter the earthquake community vulnerability levels from 1.23% to 2.8% and from 5.42% to 13.46%, respectively. Although only 11% of foreign residents are prepared for any disaster, there is a high sense of interaction amongst them and Japanese nationals, which almost eliminates isolation within communities. This study therefore proposes the utilization of some of these attributes in mobilizing specifically targeted evacuation procedures, management of evacuation centers, and disaster risk information dissemination.

## 1. General Introduction

The dynamics of the world’s population continues to evolve, and as studies show, mortality, fertility, and migration rates are the key factors driving population change in the world [1]. Population, from the perspective of diversity or dynamism, expresses “changes in the numbers, age and class distribution, sex ratio, and behavior of a population through time and space, determined by inherent characteristics of the individuals and mediated by environmental conditions, food resources, and interacting biotic agents” [2]. With fertilities and mortalities already falling in many countries [3], migration is said to be one of the major factors changing population composition in many countries such that increasing migration is significantly impacting demographic patterns and labor development [4]. However, the United Nations (2017) predicts that the contribution of migration to an entire population change is likely to increase [5]. Hence, the International Migration Report (2019) estimates that the number of international migrants is growing rapidly, from 173 million in 2000 to an estimated 272 million in 2019 [6]. This change in numbers and composition is regrettably happening at a time when the world is experiencing unprecedented natural and man-made disasters, which are extensively stretching coping mechanisms due to many obstacles [7].

The increase in disasters, changing population mix, and overwhelming coping demands are what have characterized the efforts of managing risks and vulnerabilities in the recent COVID-19 pandemic challenges [8,9]. Efforts towards managing the COVID-19 pandemic across different needs and demands bring to light the issue of at-risk or vulnerable people vis-à-vis other “normal” people in crises or disaster management such that the inability to identify, plan, and execute inclusive measures aggravates vulnerabilities and consequences. Therefore, many countries experiencing shifting population patterns/compositions, as well as increasing disasters, are facing the dilemma of initiating disaster countermeasure plans that are inclusive and meet the needs and aspirations of all parties. One such country is Japan, which per its initiative to reduce vulnerabilities and deaths to zero from disasters, is faced with the burden of disaster preparedness planning that includes the exponential rise in the number of non-Japanese nationals.

Japan, since its reforms in the 1980s to diversify its homogeneous society [10], has been overwhelmed over the years with the influx of foreign migrants [11]. Foreign residents in the country are estimated to have reached 2.93 million at the end of 2019 [12], and reports suggest that the number of foreign visitors to the country was up by 2.2% in 2019, representing a total of 31,882,100 [13]. The discussion of the numbers and composition of foreign residents in relation to the disasters in the country came to light after the 1995 Great Hanshin-Awaji earthquake, where non-Japanese nationals suffered greater casualties and property destructions per ratio than Japanese nationals [14]. This reiterated the concerns and a subsequent inclusion of foreign residents with the likes of infants, the elderly, physical and mentally handicapped people, as well as the sick, as the most vulnerable groups at risk to disasters in Japan [15]. Earlier studies have analyzed these vulnerabilities from the perspective of language abilities, disaster experience, and perception [16,17,18], which have yielded improvements in policies and initiatives accordingly. Nevertheless, recent studies still portray a persistent vulnerability of foreign residents in Japan. The need for reconsideration and the call for broader perspective of the extent of these vulnerabilities have however been magnified by the increasing number of foreign nationals and the potential risk of the anticipated Tonankai-Tokai mega earthquakes. The 2005 Hurricane Katrina in the USA gives a vivid experience of some expectations of an encounter between migrants and mega-disasters [19], which must be avoided should the mega earthquake happen in Japan. Such a scenario, and the earthquake in Haiti in 2010, suggest that spatial locations coupled with other population traits jeopardize or enhance vulnerabilities of at-risk populations, especially migrants [20,21]. This instance may specifically be important in Japan’s case, since according to [17], the proximity of many international students’ dormitories and residences to Aoba city in Miyagi prefecture was crucial to the smooth evacuation processes during the Great East Earthquake in 2011. On the contrary, a study [22] also emphasizes the challenges encountered during that same period because of the scattered locations of foreign nationals. Could it be that identifying the spatial connections or locations of foreign residents offers better insight into understanding the vulnerabilities of foreign nationals to disasters in Japan? How much of this process could reduce some of the identified challenges to disaster risk reduction? To further explore these issues, this study adopts the Tokyo metropolitan area as a case study due to the record number of foreign residents in this region as compared with other areas of the country.

## 2. The Tokyo Metropolitan Area

**Figure 1:**
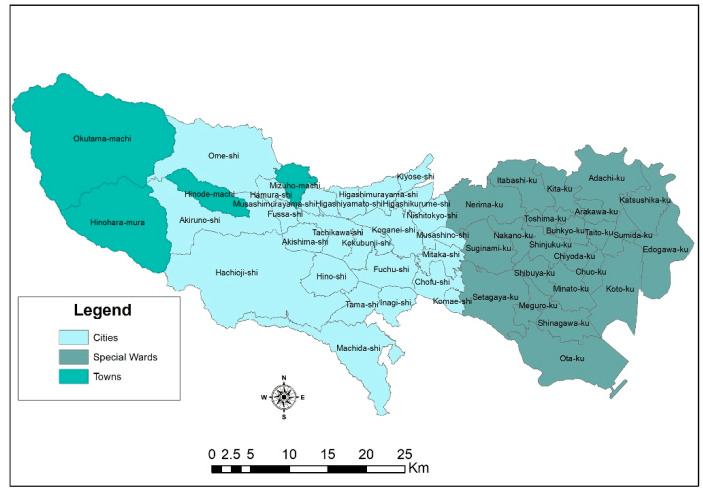
The study area: location of Tokyo metropolitan area.

The Tokyo metropolitan area (TMA) is made up of 23 special wards (区, ku), 26 cities (市, shi), 3 towns (町, machi), and 1 village (村, mura), as shown in Figure 1. It is located in the Kanto region (関東地方, Kantō-chihō) on the Honshu island of Japan. It is in the Greater Tokyo Area, which comprises other prefectures, such as Gunma, Tochigi, Ibaraki, Saitama, Chiba, and Kanagawa. The number of foreign residents in January 2010 was 577,329, which is more than 98% of other areas, such as Iwate, Yamagata, Miyazaki, and many others.

Contrary to the federal governance structure in places such as Germany, where disaster prevention and response highly rests on the state [23], Article 92 of the Japanese constitution provides regulations concerning organization and operation of local public entities, which are established under the “Local Autonomy” law. The local autonomy law establishes and separates the procedures and activities of national and the prefectural/municipal (herein referred to as “local government”) authorities. The national government is responsible for policies and affairs that require nationwide uniformity, while local governments are responsible for the day-to-day administration of settlements from towns to cities. The administration of local governments encompasses the handling of wide-scale regional affairs, communication and coordination, education and environment, and many others [24]. The Basic Act on Disaster Management Act No. 223 of 15 November 1961 also reiterates the roles and responsibilities of local governments in disaster management. This makes it efficient for disaster communication between residents and local authorities because, as pointed out by studies [25], local level efforts in disaster management are the core component of risk reduction. Hence, the Tokyo Metropolitan Government is in charge of all activities within its jurisdiction.

## 3. Disasters and Vulnerabilities of Foreign Residents in Japan

It is established that certain elements potentially underline how the population of foreign nationals are distributed in Japan [26]. However, one component that converges many elements concerns the high risk of disasters in Japan [27]. The country’s location in the “Ring of Fire” makes it vulnerable to constant volcanic and earthquake activities as well as other disasters, such as typhoons, torrential rains, heavy snowfalls, and tsunamis [28]. Although foreign nationals are distributed across the country, studies show that they are one of the most vulnerable groups of people irrespective of where they are in the country [16,27,29]. Amongst other things, low awareness of evacuation sites and procedures, limited knowledge on hazard and risk maps, and low participation and experience in disaster prevention activities characterize the vulnerable capacities of most foreigners in Japan [30]. However, what is prevalent is that most studies concerning the vulnerabilities of foreigners to disasters in Japan largely focus on issues of language barriers [17,18], diverse cultures, and perceptions [31,32], as well as the changing intensity and magnitude of disasters in the country [25]. Still, further studies have reiterated or given reservations to some of these challenges as the sole reasons. For instance, studies [16,27] acknowledge high Japanese language proficiency levels amongst some non-Japanese nationals to the extent that their level of information accessibility and processes of disaster knowledge exhibit similarities to Japanese nationals; therefore, it was concluded that not all non-Japanese nationals may be lacking Japanese language proficiency. Similarly, another study [33] emphasized the abundance of disaster prevention information for non-Japanese nationals but added that there is a lack of local content to enhance actions in the event of disasters. Furthermore, culture clashes have been evidenced to continuously exist in many disaster shelters during disaster situations, hence hindering the evacuation shelter management [17]. Some key elements from these few opinions may still highlight certain issues of language barriers, limited knowledge, and cultural differences. However, vulnerability is described to include all “conditions determined by physical, social, economic and environmental factors or processes, which increase the susceptibility of a community to the impact of hazards” [34]. Hence, the study “Disaster Mitigation: The Concept of Vulnerability Revisited”, emphasizes the need to rethink frameworks of disaster such that the way we think about disasters influences where we look for solutions [35]. As echoed by other studies, making judgement about risk is not simple for individuals or any member of the public because one must consider the nature of the hazards, the potential occurrence, the impact, as well as the assessment of a range of alternative actions and the consequences of each possible alternative [36]. It is in line with this that some studies itemize social and physical conditions of communities as two major attributes of disaster vulnerabilities such that their identification and assessment provide better alternatives to reducing risks to disaster. Living attributes of people, social networks, beliefs, customs, and other population characteristics depict social conditions [37], whilst conditions of infrastructures, physical accessibility, and densities highlight the physical aspect [38]. Consequently, approaches to analyzing these conditions have also toed similar paths by categorizing their indicators into social and built environments [39,40].

In a bid to analyze vulnerabilities within municipalities in Norway, Holand et al. (2011) asked two important questions: “who are vulnerable?” and “where do they live?”. These are legitimate questions because one cannot effectively plan, allocate resources, or understand the severity of a problem if those in need of the assistance cannot be identified. The social indicators adopted by that study to analyze the first question aligned with previous studies by [39] include race, age, educational levels, and gender amongst others. The indicators for the latter question also referenced [41], which placed emphasis on urban densities [42]. The relevance of density is expressed as “regions with a large portion of children, elderly, less educated, and ethnic minorities who tend to be more vulnerable to natural disasters, because they seem to have less physical and mental capacity to cope with them” [40]. Therefore, amongst the thirty-six indicators identified for urban vulnerability assessment, Selas and Yepes (2019) identified population density as the most important criterion [43]. However, literature reviews of previous studies on the vulnerabilities of foreign residents in Japan show characteristics that may be likened with a quest to answering the question of “who are vulnerable?”. That is, their attributes share indicators similar to those in social vulnerability studies [37,39,42]. This leaves little information regarding the question “where do they live?”.

Therefore, further studies may be useful to broadening the scope and the understanding of the challenges of foreign residents in coping with disasters in Japan. As evidenced in other studies, it is important to identify and highlight elements whose interest would be paramount to collective resilience [44]. This study aims to contribute towards closing this gap. It seeks to understand where foreign residents are living and what risks could be associated with it. This study then discusses the opportunities and constraints to reduce the possibility of vulnerabilities to disasters. This is a comprehensive multiple approach that combines both socio-economic and bio-physical elements required to understand urban vulnerabilities [45]. The results would be useful in helping the Tokyo Metropolitan Government in planning effective countermeasures since precise areas of concern are important to risk information dissemination, evacuations, and response approaches, as well as many others that are essential to avoiding the challenges that ensued during the Great East Earthquake in Aoba Ward in Sendai [17].

## 4. Existing Approach and Selected Methodology

This study utilized population distribution to analyze vulnerabilities by taking into account two residential segregation indicators. The first indicator was residential exposure (which gives characteristics of interactions amongst populations), and the second constituted residential clustering (that shows the population in reference to space per area). The output of these indicators provided both aspatial and spatial attributes of the population in relation to the areas where they live. From here, vulnerability analysis was conducted using the residential cluster attributes as one variable and adopted community risk indicators. The results are discussed in reference to other characteristics and primary data analysis collected through a survey.

### 4.1. Residential Segregation Indicators

Residential exposure is “the degree of potential contact, or the possibility of interaction, between minority and majority group members within geographic areas of a city” and is measured by diversity and isolation indices [46]. The diversity index shows the “average difference between a unit’s group proportions and that of the system as a whole“ whilst the isolation index “provide some measure of the probability that a member of one group will meet or interact with a member of another group” [47]. These are relevant in this study in that they give account of the proportion of non-Japanese to non-Japanese and to the Japanese population. They also give indication if there is isolation among foreigners to the Japanese population. These indicators elicit minority–majority group patterns relevant for policy interventions to deal with migrants–nationals integration, socio-economic inequalities, housing markets regulations, infrastructure, and service development amongst others [48,49,50,51,52]. As these traits exhibit similarities to the foreigner–Japanese integration program under the Japan Multicultural Coexistence initiative [17], they become the appropriate measures to understand more of the risk knowledge gathering through participation and interactions within communities. Equation (1) shows how the diversity index is calculated.

(1) $$h=-\overset{k}{\underset{j=1}{\sum }}P_{ij\;}log\left(P_{ij}\right)$$

where *k* represents the number of nationalities, including foreigners and Japanese, and *P_ij_* = the proportion of the population of the *j*th nationality in city *i*. This proportion is calculated by $\frac{n_{ij}}{n_{i}}$, where *n_ij_* represents the number of the population of the *j*th nationality in city *i*, and *ni* is the total number of the population in the city. The higher the *h* value, the more diverse the area [53].

The formula for calculating the isolation index is also presented in Equation (2).

(2) $$E=\sum \left(\frac{n_{fn}}{N_{fn}}\right)\left(\frac{n_{jn}}{n_{tp}}\right)$$

where $n_{fn}$ represents the number of a specific foreign national (e.g., Korean or Indian) in the city, $n_{jn}$ is the number of Japanese nationals in the city, and $N_{fn}$ represents the total number of all foreign nationals in the city. $n_{tp}$, on the other hand, represents the total number of the population in the city. If expressed in percentage, it would indicate how many foreigners out of one hundred are likely to encounter or interact with a Japanese national. A high percentage represents good interactions, while a low percentage means there is a level of isolation amongst foreigners from the Japanese citizens.

Population data of foreigners and Japanese citizens used for these analyses were collected from the data portal homepage of the Official Statistics of Japan (https://www.e-stat.go.jp/en accessed on 15 October 2020) and the Tokyo Metropolitan Government Statistical Division’s homepage (https://www.toukei.metro.tokyo.lg.jp/tnenkan/tn-eindex.htm accessed on 17 October 2020). The results are aspatial attributes that do not include the geographical area in the measurement. However, the outputs were saved in Microsoft excel 2019 (a client software by Microsoft, in Washington, DC, USA) and exported to ArcGIS 10.4 software (a Geographic Information system (GIS) application by ESRI in Redlands, CA, USA) for visual depiction.

Based on the residents register from the Tokyo Metropolitan Government, the identified nationalities are Chinese, South Korean, Vietnamese, Filipino, Nepalese, Taiwanese, American, Indian, Myanmarese, Thai, and Japanese, and other foreigners are grouped as “others”. The results from these analyses were saved in Microsoft excel and exported to ArcGIS software.

### 4.2. Residential Clustering

Clustering was identified by the density of foreign residents within 1 km x 1 km of the population grid acquired from the data portal homepage of the Official Statistics of Japan (https://www.e-stat.go.jp/en accessed on 15 October 2020). Buffer analysis was then conducted in ArcGIS to measure residential concentration radius.

### 4.3. Vulnerability Assessment and Data Source

The spatial vulnerability assessment was conducted using data from the 8th Community Earthquake Risk Assessment Study report (2018) by the Bureau of Urban Development, Tokyo Metropolitan Government (地震に関する地域危険度測定調査|東京都都市整備局 (tokyo.lg.jp) accessed on 2 December 2020). The assessment report ranks communities vulnerable to earthquake impact in the Tokyo area based on three indicators of risk on a scale from 1 (low) to 5 (high). The indicators include the risk of building collapse, buildings at risk of fire outbreak, and the degree of difficulty for emergency response in event of an earthquake. This study combines these scenarios and subsequently identifies the total earthquake impact and potentially vulnerable areas based on the risk levels. This risk assessment, however, excluded the population component in the calculation, but if this scenario is to be aligned with similar case study analyses then the proportion and type of people living in the areas should also be estimated [54]. Therefore, to understand how the living areas of foreign residents would contribute to the earthquake risk, the option would be to re-run the assessment again by incorporating the population element. However, the weighted sum overlay method in ArcGIS offers a new option where multiple analyses can be overlayed to create a new set of outputs. This is achieved by assigning weights to the variables involved based on known criteria [55]. Hence, a weighted sum analysis was conducted after obtaining the GIS shapefile of earthquake risk assessment categories from the Tokyo Government Bureau of Urban Development homepage (https://www.toshiseibi.metro.tokyo.lg.jp/bosai/chousa_6/home.htm accessed on 2 December 2020).

#### Method of Analysis

The weighted sum method was adopted for this study because it is a multi-criteria decision-making tool, which is used for decisions and problems involving multiple variables and criteria [55]. It is based on the formula as depicted below:

(3) $$WS=\overset{n}{\underset{i=1}{\sum }}W_{i}\times V_{i}$$

where SW is the weighted sum, *W_i_* is the assigned weight of the *i*th evaluating factor (representing the assigned weight of the earthquake risk impact and the residents’ population density), *V_i_* is the score of the *i*th evaluating factor (that is, within the rank 1 to 5 from risk and density), and n is the total number of evaluation factors (this time looking at the risk impact and the foreign residents’ population density). Studies have suggested that depending on the purpose of study, the number of evaluating factors is important [56].

This process works at the pixel level, so in ArcGIS software, it is incorporated into the spatial analyst environment. It has the “ability to weight and combine multiple inputs to create an integrated analysis by multiplying the designated field values for each input raster by the specified weight. It then sums (adds) all input rasters together to create an output raster” [57].

The weighted sum in the geographic information system (GIS) environment works with raster files, so the 1 km x 1 km population density shape file and the earthquake risk categories shape files are converted to a raster file. In the weighted sum environment, total weight assigned to variables (representing each raster dataset) per percentage is 100. Since there are three variables already used in the earthquake risk assessment, the population density is added as the fourth variable. By this, 0.25 weight is assigned to the density map and 0.75 weight is assigned to the risk assessment map. The figure below explains this process further.

**Figure 2:**
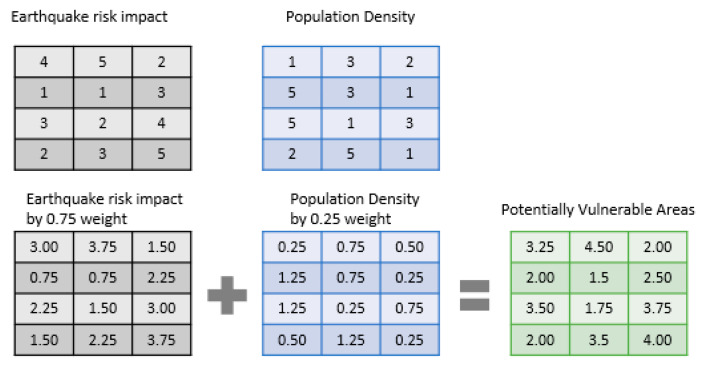
Methodology for the weighted sum.

This is a process where raster cell values are multiplied by their weight factor and the results are added together to create a new output raster. The population grid categorizes densities from 1—low to 5—high, in the same manner as the 1—low to 5—high categories in the risk assessment. As seen in Figure 2, each cell represents a pixel containing a number assigned to represent the level of earthquake risk impact or the density of foreign residents in the area. By multiplying the weights by their cell number, a new area is created. These are then summed to create the final raster representing all variables. A higher cell value in the resulting raster represents areas with high population density and high-risk value. By virtue of high-risk indication and the difficulties associated with foreigners in disaster situations from existing studies, the high-risk values in the resulting output file represent a potentially high vulnerability.

### 4.4. Empirical Data Collection and Analysis

The empirical data used for this study were gathered using online questionnaire administration on the basis that nearly 85% of all foreigners in Japan are in the working-age population between 14 and 64, distributed in different areas, and engaged in many activities [58]. These, together with the current COVID-19 restrictions, pose limitations to conducting face-to-face or in-person data gathering. Hence, as outlined by Pete Comley and Jon Beaumont (2011), using online surveys curtails these identified obstacles by offering a sense of openness, increasing geographical scope, reducing cost, and expediting responses and feedback [59]. Therefore, a link to an online questionnaire on some attributes and characteristics to enhance disaster preparedness and evacuation protocols of foreign residents was created in October 2020, shared with acquaintances through emails, SNSs, and other platforms. These were answered only by foreign residents after an initial pre-testing. At the end of the survey, a total of 106 respondents were sorted and processed within SPSS 16.0 software (a statistical software by IBM, New York, NY, USA).

## 5. Results and Discussion

### 5.1. Population Changes and Unique Locational Preferences

**Figure 3:**
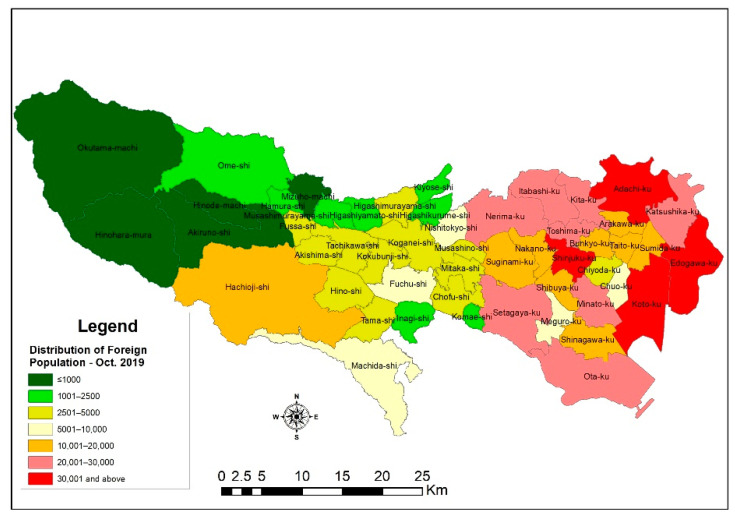
Distribution of non-Japanese nationals in the Tokyo metropolitan area.

Foreigners in Japan constitute a less significant ratio compared to other areas, such as the USA, Australia, and Europe. Still, figures gathered in this study suggest that the number of foreigners in the country has increased significantly in recent years and has been characterized by the increasing diversity of composition. The Tokyo metropolitan area had over half a million foreigners as of June 2019, and the top ten countries with high migrant populations are China, Republic of Korea, Vietnam, Philippines, Nepal, Taiwan, the USA, India, Myanmar, and Thailand, respectively. However, previous studies indicate that migrants often conglomerate in their new destinations, and the choice of location is fueled by many conditions, including path dependence [60], social network [61], and many others. Hence, Figure 3 shows that the vast majority of foreign nationals are found to reside in the eastern section of TMA, but the number dwindles as it moves towards the western side of the metropolis. Amongst the notable areas are Edogawa city, Koto city, Adachi city, and Shinjuku city, with a foreign population of over thirty thousand. The eastern sections of the metropolis also represent the 23 special wards of the TMA, and, as such, a study [62] states that the area’s unique characteristics constitute some reasons for the destination choice of many foreign populations.

**Figure 4:**
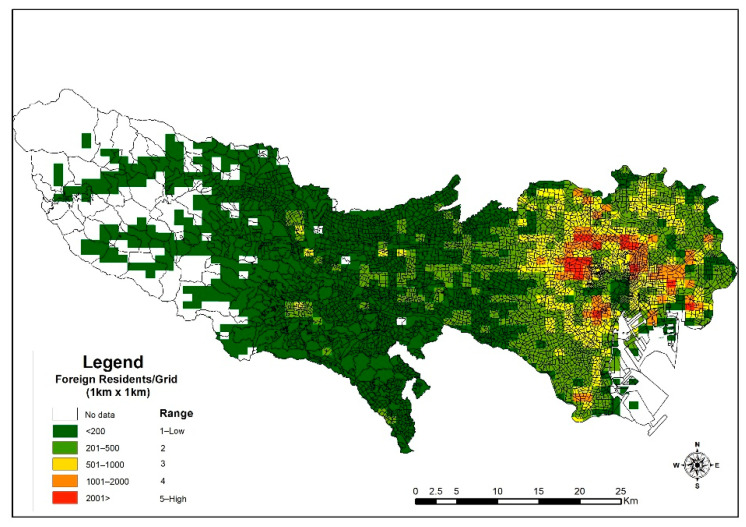
Population density of non-Japanese.

As explained further by Fahey et al. 2019, migrants face numerous challenges, such as language barriers, discrimination, culture shock, limited access to certain resources, opportunities, and services, in their host countries [49]. These make it tough for migrants to have or enjoy the best of their stay in the country. However, these challenges gradually diminish or become minimal to subsequent migrants as a result of earlier migrants who, over time, became acclimatized; gained experiences; and, through social network elements, are able to offer a conducive environment for newcomers to fit in. Therefore, new migrants are “incentivized to settle in neighborhoods that are geographically proximate to people of the same national background” and are attracted to, through cultural, ethnic, and other social connections, areas that give them the necessary assurances [49]. Many scholars then argue that these actions cause segregation or isolation of migrants from the communities they live in, and the consequences of such may be enormous [63]. The results from this study give a glimpse of this possibility in the TMA. As depicted in Figure 4, the concentration of non-Japanese nationals can be found in the eastern part of the metropolis, with a high density within a radius of about 7 km of Minato city. From a disaster management perspective, population concentration can be both advantageous and challenging. That is, the concentration of Syrian refugees in Turkey aided the study [54] in estimating potential casualties should earthquakes occur in the southeastern provinces in Turkey near the Turkey–Syria border. Based on that, various measures were proposed.

**Figure 5:**
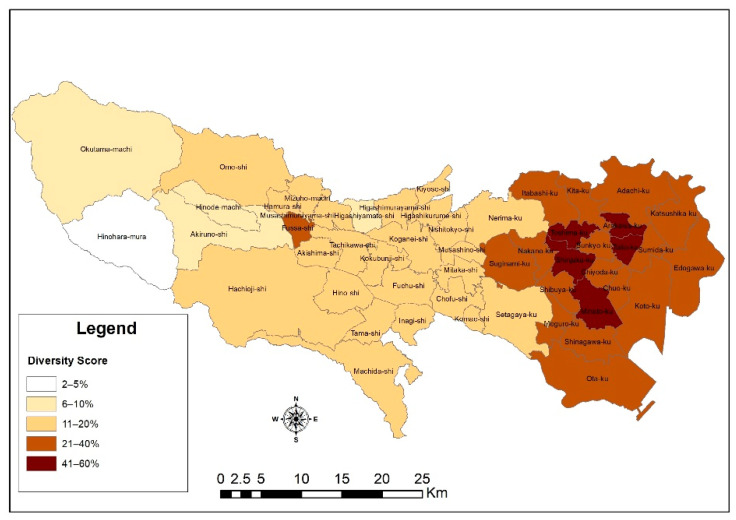
Diversity within cities in the Tokyo metropolitan area.

On the other hand, for the purposes of promoting an inclusive society as championed by many [64], minority, vulnerable, or migrant groups may be distributed evenly, but there could be a chance of isolation within them and other groups or the indigens. These elements are important for community engagement activities for disaster preparedness and prevention such that communities engage each other in disaster drill exercises, mutual help, and assistance capacity assessment, as well as inclusive community planning. Therefore, an iota of isolation may become detrimental to such causes because as evidenced by studies, self-help and mutual assistance were some key features for sustainability during the Great Hanshin-Awaji earthquake in 1995 [65]. The depiction of the results of the diversity index from the analysis, as shown in Figure 5, conforms with earlier statements that the non-Japanese population is skewed to the 23 special wards. From the results, there is an overall average of 20% diversity score or spatial distribution across cities in the metropolitans. The foreigner–Japanese population mix shows that in some areas, like Hinohara, the population composition is very homogenous with a diversity score of an average of 2.5%. However, cites like Shinjuku, Minato, Toshima, Arakawa, and Taito are highly diversified, with an average score of more than 50%. This may suggest that in some cities, more effort may be required to improve the resilience of foreign residents to disasters than that for others.

Nevertheless, the isolation index, as explained above, offers further interpretation of the diversity and how approaches and measures for disaster preparedness could be inferred to be progressing. From the analysis of Equation (2), the isolation index reveals that, on average, there is a 95% probability that a foreigner (non-Japanese) would interact with or meet a Japanese national. This gives the indication that although the diversity in some cities may be low or high, there is still the possibility of interactions and encounters. These are vital in the event of disasters and preparedness.

### 5.2. Disaster Vulnerability and Locational Preferences

**Figure 6:**
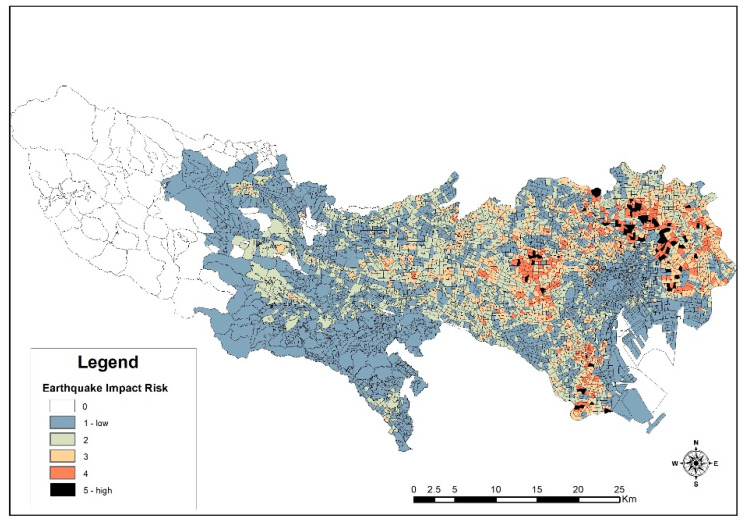
Earthquake impact risk assessment. Source: based on data from the Bureau of Urban Development, Tokyo Metropolitan Government (地震に関する地域危険度測定調査|東京都都市整備局 tokyo.lg.jp (accessed 2 December 2020).

A critical component of preparing or managing disasters is to understand the modus operandi of the available risks as prioritized in the Sendai Framework for Action 2015–2030. Therefore, the Headquarters for Earthquake Research Promotion, Japan, after a series of analyses, concluded that the next 30 years presents a critical moment in the country, which requires urgent attention, planning, monitoring, and evaluation. That is, there is a 70% possibility that a large inland earthquake would hit the southern Kanto area, with an epicenter directly below the metropolitan area [65]. Based on this scenario, its impact on buildings and the outbreaks of fires from the impact, in addition to envisaged difficulties of management authorities to curtail the situation, have since 1975 been analyzed and assessed every five years to generate an impact assessment on communities across the TMA. The eighth assessment examined 5177 communities in Tokyo, and gives the vulnerability of communities per the impact in most urbanized districts of the metropolis as reproduced in this study by the map, as shown in Figure 6. It is clear that the north-eastern and western section within most of the 23 special wards may suffer the most. The reasons assigned to areas exhibiting high risk include the presence of several wooden houses, alluvial lowlands, and narrow road networks amongst others.

By calculating the areas attributed to the various risk category levels in Figure 6, this study establishes that the low-ranked category (rank 1) constitutes the highest with about 55% of land area, whilst rank 5 represents the lowest, with 1.23% of land area. This indicates a proportionally low vulnerable area compared to areas that seem safe. However, the report indicates that rank 5 has 85 buildings per hectare at risk of collapse [66]. This notwithstanding, measures are already in place to make the city resilient to disasters, including the retrofitting of properties and infrastructures with enhanced seismic resistance material and technology [67].

A number of case studies compiled by some studies [68] express the need to highlight or pay important attention to migrant population, risk, and disaster preparedness activities. This assertion stems from the fact that limited language proficiency, limited knowledge of their destination’s hazards, laws, discrimination, and others become highly profound in disaster events. Besides these, behavior of migrants before and after disasters makes it important to include or recognize them as a unique entity in risk assessment [19,54]. Therefore, as evidenced in the above discussion about the clustering in certain areas of the metropolis, there is an implication of potential difficulties should a disaster occur. Thus, a clear representation of this potential vulnerability can be observed from the weighted sum results of the earthquake risk impact assessment and the foreign residents’ population grid density.

**Figure 7:**
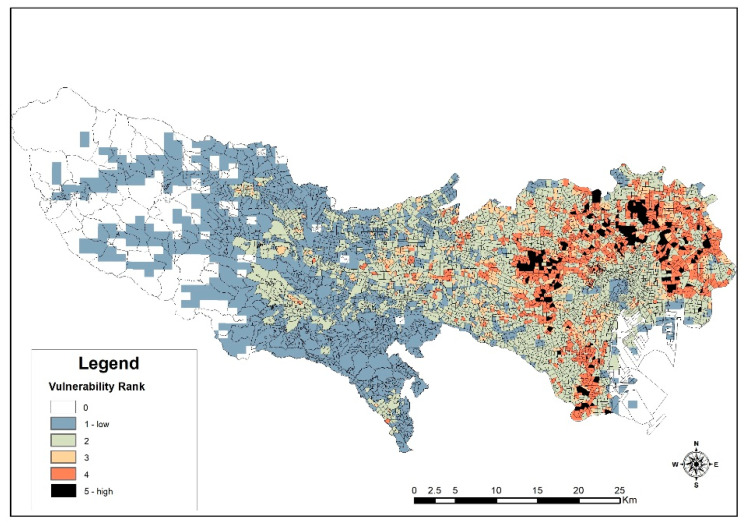
Vulnerability areas for non-Japanese.

As seen in Figure 7, the results from this combined scenario show a different output from Figure 6. That is, the radius that contains most of the foreign residents also exhibits high-risk levels. This in part can be explained by the earlier discussion on migrants’ choice of location and also highlights a point that locational attractiveness and other migrant connections supersede the obvious presence or availability of risk. Therefore, recalculating the risk areas after adding population densities of low to high with the rank categories from low to high shows changes in risk levels. From Table 1, it can be seen that the density variable alters the community vulnerability levels from the initially examined earthquake impact assessment. The area for rank level 5 increased from 1.23% to 2.8% of space, whilst combined levels of 4 and 5 rises from 5.42% to 13.46%. This indicates that should there be a mass collapse of buildings as initially suggested, many foreigners in certain regions may need further assistance. At this stage, a conclusion on these results would be inconclusive, because the earlier section discussion pointed to a promising picture of a more open society where most cities are diverse with high interaction rates. The results from the online survey shed more light on these issues.

### 5.3. Disaster Preparedness Perspectives of Foreign Residents 

Over 50% of the respondents to the survey were students, whilst the rest represents other professions. According to the Japanese Disaster Countermeasures Basic Act (Act No. 223, 15 November 1961) Article 7.2. “…. residents of an area under local government are obligated to contribute toward the cause of disaster prevention by taking their own measures to prepare for disaster and by participating in voluntary disaster prevention groups etc.”. This suggests that the guaranteed safety from disasters depends on the preparation of the individual. However, studies advocate that the approaches undertaken by people to meet such obligation depend on some qualities or attitudes of the person. A previous study [69] recognized these as risk identification, cognitive properties, emotional instinct, trust, and behavior of the person towards the risk. Therefore, the portrayal of an observed vulnerability, risk, or disaster impact may not reflect on the view of the person involved. This is evidenced from the survey as to the perspective of the recognition of risk such that although about 62.3% of respondents have experience of disasters in Japan, nearly 27% stated that they are not prepared and have no clue what to do should a disaster occur. This notwithstanding, 62% have no idea if they are prepared or not prepared, with only 11% stating that they are prepared for disasters in the country. These responses could have arisen from the fact that only 49% believe their area of residence are high disaster-prone areas, with the rest either not sure of the situation or believe there is no worry for disasters in their areas, as shown in Table 2.

Further probing into this potential lapse in preparedness also shows that in as much as they may not be prepared, there is a strong trust in information from local and national governments (89%). This is crucial if compared with the situation during the 2005 Hurricane Katrina. According to [70], the distrust of authorities, particularly from minority ethnic groups, was one of the many reasons for the lapses in response to evacuation warnings, which resulted in several casualties.

## 6. Implications

The results from this study give two indications with respect to spatial vulnerabilities and the attributes of the population. By combining earlier studies on non-Japanese nationals and disaster situations from the works of [17,22,30], the following key challenges and opportunities emerge: a) the proximity of the residential neighborhoods of foreign residents’ aid evacuation processes but b) brings challenges at the evacuation shelters due to ethnic differences and the Japanese culture, and also c) dispersing residential locations creates some challenges in disaster and evacuation management, such that d) the increasing number of foreign residents means a new framework of planning that involves the considerations of the needs of these different population mixes. However, the approach adopted and the result from this study can enhance disaster inclusive planning that addresses most of the issues above. Since this study identifies greater interactions between groups and Japanese nationals, it could be a way to enhance the Multicultural Coexistence Initiative (Tabunka Kyosei) and the Zero Information Refugee Project (Jōhō nanmin zero purojekuto), which are aimed at smoothening the disaster information transmission and the transition of non-Japanese nationals into the local communities in areas such as Shinjuku, Nakano, Arakawa, Katsushika, Adachi, and Edogawa. Additionally, special attention could be given to the vulnerable areas, which have been enhanced by the high density of foreign residents. Furthermore, this identification of clustering can offer insight into evacuation planning and management of evacuation centers. This is because each clustered area has a dominant migrant group, which can be useful when planning the language, food, and type of information to give through the utilization of some group leaders.

## 7. Conclusions

This study sought to analyze the vulnerability of foreign residents in the Tokyo metropolitan area and in Japan through the perspective of population distribution attributes. It highlights that there is indeed an increase in the number of foreign residents in the country, and it finds evidence that they are spatially clustered in some parts of the metropolis, especially within the 7 km radius of Minato city. However, there is no evidence of isolation from their Japanese counterparts, as the study finds a 95% probability that a non-Japanese national would interact with or meet a Japanese national on a daily basis. Nevertheless, this result is highly influenced by the 60% diversity in the 23 special wards compared to the very homogenous areas in the west side of Tokyo, such as Hinohara village, with a 2.5% diversity score. This notwithstanding, the mere choice for place of residence within cities may potentially increase their vulnerabilities should an earthquake occur. This is because high residential density as a potential risk factor could increase casualties or severe impacts from building collapse, fire outbreaks, and others. The densities in areas where many non-Japanese nationals reside alter the earthquake community vulnerability levels from the initially examined earthquake impact assessment in a way that areas ranked level 5 (from an earthquake risk impact scale of 1—low to 5—high), increase from 1.23% to 2.8%, whilst combined areas with levels 4 and 5 rise from 5.42% to 13.46%. In previous studies that used densities and spatial clustering in vulnerability analyses, this situation would have caused the confirmation of high-density and high-risk areas as vulnerable.

Still, empirical evidence suggests divergent opinions as to how the foreign residents see the risk around where they live, and this also influences their preparedness for a disaster in the metropolis. Only about 23% see the areas they live as disaster prone. This is also reflected in their reactions to disaster preparedness. Around 11% of our sampled respondents were able to state categorically that they are prepared should there be a disaster in the country, with the rest either unprepared or having no idea what to expect.

Although this study shows evidence of the vulnerabilities of foreign residents to potential disasters, the approach adopted makes it clear that the same attributes that make them vulnerable are to be utilized to mobilize specifically targeted evacuation procedures, management of evacuation centers, and disaster risk information dissemination. Further studies may, however, be needed to understand what draws foreigners to their choice of living areas, how disaster information and activities are shared in community interactions, and how these could be enhanced for disaster preparedness.

## 8. Limitations

It would have been ideal for the sampled population to be taken from the identified vulnerable areas to effectively correspond them with their vulnerability levels. However, the COVID-19 pandemic restrictions made it impossible.

Furthermore, the probability of interactions as depicted in this study may have limitations in terms of giving further information as to how much it can contribute to overall vulnerability in both quantitative and qualitative terms. Therefore, further studies may be required to understand how interactions are correlated to vulnerability through empirical data collection and analysis, especially in the 23 special wards of the Tokyo metropolitan area. Nevertheless, the approach adopted gives a fair idea of issues that can be utilized to reduce risk and enhance resilience to disasters in the cities.

## Figures and Tables

**Table 1:** Calculated areas of risk categories.

Rank	Earthquake Impact Vulnerability Risk		Vulnerability Risk	
	No. of Pixels	%	No. of Pixels	%
1	181,923,630	54.88%	156,577,182	43.96%
2	92,037,529	27.76%	127,079,498	35.68%
3	39,590,981	11.94%	24,574,898	6.90%
4	13,884,722	4.19%	37,715,789	10.59%
5	4,086,091	1.23%	10,224,360	2.87%
		100.00%		100.00%

**Table 2:** Perception of disaster at areas of residence and disaster preparedness.

My Area of Residence Is a High Disaster-Prone Area		Disaster Preparedness (%)			Total (%) of High Disaster-Prone Area
		Yes	Somewhat Prepared	No	
Agree		6.6	31.1	11.3	49.1
	% of disaster preparedness response	58.3	50.0	42.9	
Neither agree nor disagree		1.9	17.0	9.4	28.3
	% of disaster preparedness response	16.7	27.3	35.7	
Disagree		2.8	14.2	5.7	22.6
	% of disaster preparedness response	25.0	22.7	21.4	
Total	Total % of disaster preparedness	11.3	62.3	26.4	100

## Data Availability

Publicly available datasets were analyzed in this study. This data can be found here: https://www.toshiseibi.metro.tokyo.lg.jp/bosai/chousa_6/home.htm, https://www.e-stat.go.jp/en (accessed on 15 October 2020).
